# Refractory Unforeseen Anaphylaxis Case in a Rural OR Unit

**DOI:** 10.1155/2020/5283279

**Published:** 2020-01-28

**Authors:** Sayed Hafizi, Christine Nadeau, Mohamed Gazarin, Emily Mulligan

**Affiliations:** Winchester District Memorial Hospital, 566 Louise St, Winchester, ON, Canada K0C 2K0

## Abstract

A 35-year-old female patient with no previously documented allergies who was admitted for elective gynaecological surgery, developed rapid onset, severe anaphylaxis, with dyspnea and cardiovascular collapse, in the operating theatre after receiving routine IV cefazolin prior to induction of anesthesia. She failed to improve with two doses of intramuscular epinephrine followed by two boluses of intravenous epinephrine, but responded to an epinephrine infusion. She was assessed by Internal Medicine and discharged home the following day. This event demonstrates the speed, severity, and profound hypotension in an allergic reaction from intravenous medication, challenges in managing anaphylaxis, and importance of prompt administration of epinephrine via IM route, followed by IV if necessary, in the OR. The case highlighted the inability to ascertain the causative agent through typical allergy testing.

## 1. Introduction

Assessment and management of serious allergies is a key component in patient safety in all clinical environments. An accurate and detailed allergy history is the standard of care, and in most cases, prevents the rare occurrence of anaphylaxis and its associated morbidity and mortality. However, despite our very best efforts, some sentinel events can occur which call into question the mechanism behind certain drug reactions, and require critical care to stabilize an affected patient. This particular case was striking in the speed of onset, severity of symptoms, the known history of previous cephalosporin administration without adverse reaction, and subsequent negative allergy test results.

## 2. Case

The patient was admitted into day surgery at Winchester District Memorial Hospital for an elective posterior vaginal repair. Her past medical history was significant only for a skin and soft tissue infection treated safely with PO cephalexin in 2013, and for an upper respiratory tract infection treated with amoxicillin in December 2016; surgical history was significant only for an uneventful total hysterectomy and tension-free vaginal tape (TVT) to correct a vaginal prolapse in 2016, during which she had received prophylactic intravenous (IV) cefazolin without adverse reaction. The patient had a documented incident of adverse reaction to ciprofloxacin, which caused nausea and vomiting.

As summarized in Table 1, the patient was feeling well at the time of admission and had followed preoperative fasting instructions. A routine infusion of Ringer's lactate was initiated. After the anesthetist had reviewed the patient's history and examined the patient, she was transferred into the operating theatre where the team was waiting. The patient's preoperative vitals were as follows: blood pressure (BP) 111/94 mmHg, heart rate (HR) 54 beats per minute (bpm), and oxygen saturation (O2Sat) 97%. Monitors were applied and the “time-out” was performed. Intravenous infusions of cefazolin 2 g and midazolam 2 mg were initiated. Approximately 2 minutes after the medications began infusing, the patient stated she felt a sense of doom and was itchy. In the time it took to ask her where she was uncomfortable, she had become deeply flushed and was in respiratory compromise. The patient then lost consiousness. Within seconds she received a first dose of 0.4 mg intramuscular epinephrine but became profoundly hypotensive nonetheless. Patient vitals at that time were BP 70/45 mmHg, HR 115 bpm, and O2Sat 80%. A code blue was called when her pulse became faint and the team began resuscitation measures. The patient's pulse returned before chest compressions were needed. Her airway remained patent with absence of obstructive symptoms and she was able to be ventilated using bag-valve mask ventilation (BVM). Diphenhydramine, ranitidine, dexamethasone, two liters of crystalloid, and an additional dose of 0.4 mg intramuscular epinephrine were all administered in the following minutes, followed by two boluses of 5 mcg intravenous epinephrine and salbutamol via BVM.

After approximately 3 to 4 minutes of unresponsivness the patient regained consiousness, maintained her airway, and was able to speak. She reported feeling weak and that her face was swollen. On auscultation, there was no significant wheezing and no hives were noted on her body. Investigations revealed a normal electrocardiogram (ECG), complete blood count (CBC), electrolytes, renal function, and noncontributory chest X-ray. She stabilized clinically over the following 16 hours and was discharged the following day.

## 3. Discussion

Anaphylactic reactions are quite rare in perioperative settings. According to studies conducted in Europe, Australia, and New Zealand, one out of every 4,000 to 20,000 patients experiences a perioperative anaphylactic reaction [1–8]. Neuromuscular blocking agents, latex, and antibiotics account for the majority of perioperative anaphylactic reaction triggers [9].

Of all perioperative anaphylactic reactions, antibiotics cause approximately 15% [10]. Cefazolin is an example of a cephalosporin antibiotic commonly used in perioperative settings to lower the risk of postoperative infections [11]. It exerts its effects by inhibiting bacterial cell wall synthesis [12]. Some individuals activate an immune response against cefazolin as their immune systems identify it as a foreign invader or allergen. In turn, the body increases the number of IgE antibodies against the specific allergen. In addition, the IgE antibodies also prime mast cells and basophils, thus triggering the release of histamine, tryptase, and other molecules. Once these molecules are released, a cascade of events are actuated including the inflammatory response, bronchoconstriction, and increased mucus secretion—all of which were present in the patient [9] (Table 1). Cefazolin-provoked anaphylaxis, although rare, has been noted to occur in some patients [1, 13–16]. Anaphylactic reactions caused by cefazolin can be very severe and result in death 3% to 9% of the time [1, 17, 18]. For this reason, extreme care must be taken to prevent such occurrences.

The patient's clinical symptoms support that she had suffered an anaphylactic shock. Since the patient had undergone previous surgery with similar preoperative procedures with no notable adverse reactions, it was almost impossible to predict that such a life-threatening reaction would occur during this procedure. Moreover, the patient's medical history reveals that she safely received PO cephalexin in 2013 and IV cefazolin in 2016.

After the anaphylactic incident, the patient underwent intradermal allergy testing for multiple agents including major and minor penicillin determinants. The major determinant antigen contained penicilloyl-polylysine whereas a minor determinant mixture was composed of benzylpencillin, benzylpenicilloate, and benzylpenilloate. The patient was also tested for cefazolin, latex, midazolam, and chlorhexidine allergies. Interestingly, the patient did not elicit a positive reaction to any of the tested agents; therefore, the allergy consultant was left to determine the causative agent based on probability. Because midazolam was statistically unlikely to be the causative agent, the most likely reason for the anaphylaxis was determined to be cefazolin.

According to one study by Romano et al., 13 out of 76 adults who displayed immediate reactions to cephalosporins had received negative results to all cephalosporin allergologic tests. After eight of the 13 subjects consented to be challenged and reevaluated, two (25%) who initially tested negative reported positive [19]. It is important to note that there are three major challenges with cephalosporin skin testing: (1) there are several cephalosporin allergenic determinants that have not been conclusively defined and understood, (2) cephalosporin-protein conjugate reagents for testing are not commercially available, and (3) the negative and positive cephalosporin testing predictive values are not well established. Therefore, a negative response to the test means that the patient could have been allergic to a metabolite or to a metabolite-protein complex [20]. Thus, such a result in our case must be interpreted cautiously and cannot be used to deem cefazolin as a noncausative agent for the anaphylactic reaction [21, 22].

Since allergy testing for cephalosporins may be insufficiently sensitive, procedures such as basophil activation test and/or oral provocation tests may be carried out to verify the drug responsible for the adverse reaction [23]. Unfortunately, neither of the two tests were conducted in our case.

Looking back at the case, we realize that it may have been useful to obtain a serum tryptase level within 15 minutes to 3 hours after onset of anaphylaxis symptoms and then 24 hours after all signs and symptoms had resolved. Elevated levels of mature or total tryptase in serum may have been useful for differentially diagnosing anaphylaxis from other conditions such as systemic mastocytosis, vasovagal reactions, or septic shock [24].

This case highlights the severity of anaphylactic reactions and the importance of a proactive healthcare team. The patient had a severe anaphylactic shock to cefazolin which was successfully treated due to the immediate and aggressive response by healthcare providers. In this regard, mock drills for intraoperative emergencies, such as anaphylactic shock, would be beneficial to practice recognition and the skills required to react quickly and appropriately. In our case, the team felt that having an anesthetist with an extensive emergency room experience played a role in the swift recognition and handling of the case. Furthermore, this case also illustrates the possibility of any patient presenting a false negative result to cephalosporin allergy testing. As a result, healthcare providers must interpret such results cautiously and remain proactive to prevent and treat anaphylactic reactions. This patient was otherwise healthy and had once previously received cefazolin in an operative setting without any adverse reaction.

## Figures and Tables

**Table 1:** Timeline of events relevant to patient's admittance to day surgery and anaphylactic reaction.

Timeline of events	
12:15	Patient preop vitals BP 111/94 mmHg, HR 54 bpm, and O2Sat 97%. Patient was brought into the operating theatre, feeling well. Monitors applied to patient, “time-out” done. Cefazolin 2 g IV infused, followed by midazolam 2 mg IV.

12:17	A few seconds after midazolam was initiated, she reported a feeling of “doom,” itching in the face and chest, followed by difficulty in breathing and loss of consciousness. Prominent flushing was noted over face and chest. Profound hypotension (BP of 70/45 mmHg) despite a first dose of epinephrine 0.4 mg IM within one minute of symptoms. Patient heart rate was 115 bpm and O2Sat was 80%.

12:18	Diphenhydramine 50 mg IV, ranitidine 50 mg IV, and dexamethasone 8 mg IV were given. 2 L fluid bolus was started under pressure. Pulse was nonpalpable for less than 10 seconds, code blue called with rapid response from OR team. The airway remained patent and pulse returned spontaneously before compressions were initiated.

12:21	Salbutamol was administered, second dose of epinephrine 0.4 mg IM given, along with two boluses of 5 mcg IV epinephrine followed by a continuous infusion. Patient regained consciousness, after approximately 3-4 minutes of absence. She continued to feel weak and reported that her face was swollen.

12:30	Received odansetron IV for nausea. The airway was continuously monitored out of concern for a need to intubate; however, it remained patent and oxygen was supplemented via nasal prongs. On auscultation, there was no significant wheezing. She improved clinically with the epinephrine infusion.
